# Geographical distribution and antibiotics susceptibility patterns of toxigenic *Vibrio cholerae* isolates from Kisumu County, Kenya

**DOI:** 10.4102/phcfm.v12i1.2264

**Published:** 2020-12-08

**Authors:** Silas O. Awuor, Eric O. Omwenga, Ibrahim I. Daud

**Affiliations:** 1 Department of Health, School of Health Sciences, Kisii University, Kisii, Kenya; 2 Kenya Medical Research Institute, United States Army Medical Research Directorate-Africa, HJF Medical Research International, Kericho, Kenya

**Keywords:** *Vibrio cholerae*, antimicrobial susceptibility, Kisumu, tetracycline, cotrimoxazole, Kenya

## Abstract

**Background:**

Multiple drug resistance has become a major threat to the treatment of cholera. Recent studies in Kenya have described the epidemiology, especially the risk factors, of cholera; however, there is little information on the phenotypic and drug susceptibility patterns of *Vibrio cholerae* (*V. cholerae*) in outbreaks that in the recent past have occurred in western Kenya.

**Aim:**

To characterise and determine the antibiotics’ susceptibility profiling of toxigenic *V. cholerae* isolates from Kisumu County.

**Setting:**

The project was conducted in Kisumu County, Kenya.

**Methods:**

A total of 119 *V. cholerae* O1, biotype El Tor, isolates collected during 2017 cholera outbreak in Kisumu County were used for this study. The samples were cultured on thiosulphate-citrate-bile salts sucrose (TCBS) agar and biochemical tests were carried out using standard procedures. Susceptibility tests were conducted by using various conventional antibiotics against standard procedures.

**Results:**

Of the 119 isolates, 101 were confirmed to be *V. cholerae* belonging to serotypes Inaba and Ogawa, with Inaba being the predominant serotype (73.95%). The isolates were susceptible to ciprofloxacin (100%), ofloxacin (100%), gentamycin (100%), doxycycline (99%), ceftriaxone (99%) and streptomycin (96.04%) antimicrobials, and resistant to erythromycin (53.47%), amoxicillin (64.4%), nalidixic acid (83.2%) and ampicillin (89.11%), with high resistance to cotrimoxazole (99%) and tetracycline (97%).

**Conclusion:**

*Vibrio cholerae* was resistant to multiple antibiotics, including those commonly used in the management of cholera. Taken together, there is a need to carry out regular surveillance on antimicrobial drug resistance during outbreaks.

## Introduction

Antibiotic resistance has been increasing since the introduction of antibiotics in the middle of the 20th century.^1,2^ Since the introduction of tetracyclines, many other antibiotics have been introduced, but each has shown resistance at some level.^3,4^ The level of circulating virulent, multiple drug-resistant (MDR) bacteria has been a global threat to healthcare.^5,6^ Toxigenic *Vibrio cholerae* (*V. cholerae*) O1 or O139 has been a cause of gastrointestinal infections and is involved in severe outbreaks of dehydrating diarrhoea in most of developing nations worldwide.^1,2^ Cholera – a disease associated with poor sanitation – is often transmitted by consumption of food and water contaminated with bacterium.^7^ Cholera re-emerged as a major infectious disease in the past, with a global increase in its incidence.^8^ In addition, there was unprecedented appearance of an epidemic strain of *V. cholerae* non-O1, classified as *V. cholerae* O139, in late 1992 in West Bengal state of India.^9^ Since 2007, Kenya has experienced cholera outbreaks characterised by increased mortality.^10^ For instance, a cholera outbreak in November 2007 that began in Nyanza claimed 67 lives out of 1243 cases by April 2008.^11,12^ Previous cholera outbreaks in Nyanza were not as severe as that of 2007.^13^ Currently, *V. cholerae* antimicrobial resistance has become a global concern as fewer new antibiotics are being discovered, with more pathogens becoming resistant to most commonly used antibiotics.^14^ Antibiotics that have been used in the management of cholera cases for decades such as tetracyclines, which include doxycycline and tetracycline, have been associated with the presence of *IncC* conjugative plasmids.^15,16,17^ In Kenya, doxycycline and tetracycline are the first-line drugs used for cholera treatment in adults. Erythromycin and chloramphenicol are used for the treatment of cholera in children and pregnant women.^18^ Despite the body of research on the epidemiology of cholera and its associated morbidity in Kenya, and Kisumu County in particular, studies focusing on the phenotypic and molecular characteristics of *V. cholerae* in outbreaks amongst Kisumu patients are limited and often anecdotal. This study is aimed to characterise and determine the antibiotics susceptibility profiling of toxigenic *V. cholerae* isolates from the various sub-counties of Kisumu County.

## Materials and methods

### Study design

This was a descriptive cross-sectional study that focussed on the 2017 cholera outbreak previously isolated from diarrhoeal stool specimens. The specimens were obtained from patients presenting with passage of three or more watery stools with or without vomiting during cholera outbreaks.

### Study area

The study was performed at the six sub-counties of Kisumu County, including Muhoroni, Nyando, Nyakach, Kisumu West, Kisumu East and Seme.

### Study samples

A total of 119 isolates of *V. Cholerae* O1, previously isolated from diarrhoeal stool specimens between January and December 2017 within the county, were used for this study. The isolates were stabilised and stored at -80 °C freezing condition at the Centre for Global Health Research.

## Study procedure

### Identification and susceptibility testing of *Vibrio cholerae* isolates

The isolates which were stored at -80 °C were revived under sterile conditions as described previously.^17^ The isolates were sub-cultured in thiosulphate-citrate-bile salts sucrose (TCBS) agar, which was prepared as per the manufacturer’s instructions. The plates were then incubated at 37 °C for 18–24 h in aerobic conditions. After 24 h of incubation, bacterial cultures giving growth of pure green and yellow colonies were presumed to be that of *V. cholerae* and subjected to Gram staining, and biochemical and serological identification testing.

### Gram staining

Thin smear was prepared from the colonies grown on TCBS media, air-dried and heat-fixed. The smear was then stained using Gram staining technique.^19^

### Biochemical identification

The isolates were subjected to biochemical identification using Application Programming Interface (API) 20 E kit as per the manufacturer’s instructions (API 20 E; BioMerieux, Charbonnieres-Les-Bains, France) together with established protocols.^20,21,22,23^ Briefly, 2 mL of API 0.85% NaCl was inoculated with pure colonies of young cultures (18–24-h old) to make a 0.5 McFarland standard and measured with the ATB densitometer (Denka Seiken Co., Ltd, Tokyo, Japan). Each cupule of the strip was dispensed with 56 microliter (µL) of suspension using ATB electronic pipette. The Arginine dihydrolase (ADH) Lysine decarboxylase (LDH) urea test (UREA) Arabinose fermentation (LARL) Ornithine dehydrogenase (ODC) Ketogluconate (KG) and 5KG tests were covered with two drops of mineral oil. The lid was placed on the strip and incubated at 36 °C ± 2 °C for 24 h (± 2 h) in aerobic conditions. One drop of JAMES reagent was added in IND reaction and the strips were read using mini API machine (BioMerieux, Charbonnieres-Les-Bains, France). The results were again read through computer and interpreted using mini API identification software (BioMerieux, France). All tests were performed in triplicate and were independent of each other.

### Serological identification

The serologic identification was conducted by the slide agglutination technique with polyvalent anti-sera for *V. cholerae* O1 and O139, and monovalents for serotypes Inaba and Ogawa (Denka Seiken Co. Ltd., Japan) according to the manufacturer’s instructions and previously published protocols.^20,21,22,23^ The confirmed *V. cholerae* isolates were further sub-cultured on nutrient agar (that was prepared as per the manufacturer’s instructions) and incubated overnight at 37 °C for 18–24 h in aerobic conditions. A pure colony was picked aseptically and mixed in a drop of sterile normal saline on a glass slide, making a milky suspension. A drop of antiserum was added onto the drop and mixed. Agglutination reaction was observed. Positive agglutination confirmed serotype and subtype of isolates. In this test, K3 and K14 strains belonging to *V. cholerae* O1 biotype El Tor, subtype Ogawa, were used as positive controls, whilst *Escherichia coli* strain ATCC 25922 was used as a negative control. All tests were carried out in triplicate independent of one another.

### Antimicrobial susceptibility testing

Susceptibility to antimicrobial agents was assayed by the disk diffusion method described previously.^20^ Drug incorporated disks were used for O/129 vibriostatic agent whereas, for the rest of the antimicrobials, their minimum inhibitory concentration (MIC) was determined by the Etest (previously known as Epsilometer test) method as used before.^21^ *Escherichia coli* standard strain, ATCC 25922, was used as internal quality control. The procedure for antimicrobial susceptibility testing was performed as per the Clinical Laboratory Standards Institute (CLSI) guidelines.^19^ All tests were carried out in triplicate independent of one another.

### Data analysis

All experiments were conducted in triplicate to validate reproducibility. Statistical analysis was performed using Stata software. Data on socio-demographics were summarised by frequencies and percentage values. All values of diameter zones of inhibition were reported as mean ± standard error. Data analysis was performed during and after collection. The study did not involve patients.

### Ethical consideration

Confidentiality and privacy were strictly adhered to and no names of individuals were recorded or made known in the collection or reporting of information. The study was granted ethical clearance by the Board of Postgraduate Studies (BPS) of Kisii University, and ethical approval to conduct the study was sought from the Institutional Research Ethics Committee (IREC) at Moi University/Moi Teaching and Referral Hospital (MTRH) and the National Commission of Science, Technology and Innovations (NACOSTI).

## Results

### Socio-demographic data of the *Vibrio cholerae* cases in the 2017 cholera outbreaks in Kisumu

Samples were obtained from patients presenting with symptoms of cholera. The mean age of the patients was 22.62 years (range: 2–73 years). The most infected age group was 5–14 years (42%), and the least infected age group was 25–34 years. In terms of gender, women (57%) were more infected than men. Geographically, the majority of cases were from Seme sub-county (20%), whilst the least (14%) were from Kisumu West sub-county (Table 1).

**TABLE 1:** Socio-demographic data, serological identification, colony morphology, Gram staining and cholerae serotypes distribution amongst the study isolants (*n* = 101).

Variables	*N*	%
**Age (years)**		
< 5	14	11.76
5–14	50	42.02
15–24	18	15.13
25–34	9	7.56
> 35	28	23.53
**Gender**		
Female	68	57.14
Male	51	42.86
**Study location**		
Muhoroni	19	15.97
Nyando	20	16.81
Nyakach	20	16.81
Kisumu East	19	15.97
Kisumu West	17	14.29
Seme	24	20.17
**Serological identification**		
Positive	101	84.87
Negative	18	15.13
**Colony appearance on TCB**		
Yellow mucoid	101	84.87
Non-yellow mucoid	18	15.13
**Gram stain reaction**		
Positive	101	84.87
Negative	18	15.3
**Cholerae serotypes distribution:**	Ogawa (*n* = 13)	Inaba (*n* = 88)
Muhoroni	3	16
Nyando	2	18
Nyakach	2	18
Kisumu East	1	18
Kisumu West	5	12
Seme	0	24

TCB, thiosulphate citrate bile.

### Morphological and biochemical characteristics of isolated *Vibrio cholerae*

Of the 119 isolates, 101 (85%) were found positive for *V. cholerae* based on a combination of serological, biochemical and cultural identification techniques (Table 1). Growth characteristics of presumptive *V. cholerae* were determined on TCBS selective media as large yellow mucoid colonies, which was suggestive of the presence of *V. cholerae* strains. The colonies also produced Gram-negative rods by Gram staining (Table 1).

Next, all presumptive colonies of *V. cholerae* were subjected to further identification based on biochemical reactions. Isolates that were Gram-negative rods, positive for Voges–Proskauer and Indole tests, and negative for hydrogen sulphide test, together with glucose, sucrose and mannitol fermenters, were confirmed as *V. cholera* (Table 2).

**TABLE 2:** Summary of biochemical tests reactions against the *Vibrio cholerae* isolates obtained from the 2017 cholera outbreaks in Kisumu County (*n* = 101).

Characteristic/result	Indole		Voges Proskauer		Glucose		Hydrogen sulphide		Mannitol		Lysine decarboxylase		Ornithine decarboxylase		Sucrose	
	Frequency	%	Frequency	%	Frequency	%	Frequency	%	Frequency	%	Frequency	%	Frequency	%	Frequency	%
**Positive**	101	84.87	101	84.87	101	84.87	18	15.13	101	84.87	101	84.87	101	84.87	101	84.87
**Negative**	18	15.13	18	15.13	18	15.13	101	84.87	18	15.13	18	15.13	18	15.13	18	15.13

Moreover, the presumptive *V. cholerae* isolates were further tested for agglutination with polyvalent O antisera and only positive isolates (84.9%) were stocked as *V. cholerae.*

### Characterisation of *Vibrio cholerae* isolates

Biochemical identification using mini-API ID 20E (Biomeriux SA, France) confirmed that 101 (84.9%) isolates were *V. cholerae* isolates, whilst 18 (15.1%) were non*-V. cholerae* isolates. Serological identification further showed that 101 (84.9%) isolates were of *V. cholerae* O1. This test further indicated that both serotypes were present, with 88 (73.95%) serotypes being Inaba and 13 (10.92%) as serotype Ogawa (Table 1).

### Distribution of cholera and cholera subtypes in the study sites

The majority of infected persons were from Seme sub-county (20.2%), followed by 16.8% from Nyando sub-county, 16.8% from Nyakach sub-county, 16.0% from Muhoroni Sub- county, 16.0% from Kisumu East sub-county, whilst the least (14.3%) were from Kisumu West sub-county. The distribution of Inaba and Ogawa between the study sites varied, with Kisumu West sub-county having the majority of serotypes Ogawa (29.41%) and Inaba (70.59%) (Table 1).

### Antimicrobial susceptibility patterns

The positive isolates for *V. cholerae* were screened for their susceptibility to various antibiotics commonly used to manage cholera cases. All 100% isolates were susceptible to gentamicin at a concentration of 0.016 µg/mL – 256 µg/mL and ofloxacin at a concentration of 0.002 µg/mL – 32 µg/mL. However, there was a decreased susceptibility to chloramphenicol (55.45%) at a concentration of 0.016 µg/mL – 256 µg/mL. In all, 10.89% of the *V. cholerae* isolates were susceptible to ampicillin at a concentration of 0.016 µg/mL – 256 µg/mL, 99% to doxycycline at a concentration of 0.016 µg/mL – 256 µg/mL, 96.04% to streptomycin at a concentration of 0.064 µg/mL – 1024 µg/mL and 2.97% to tetracycline at a concentration of 0.016 µg/mL – 256 µg/mL. It was observed that 64.36% of *V. cholerae* isolates were resistant to amoxicillin at a concentration of 0.016 µg/mL – 256 µg/mL, 98.02% to cotrimoxazole at a concentration of 0.016 µg/mL – 256 µg/mL, 53.47% to erythromycin at a concentration of 0.016 µg/mL – 256 µg/mL, 97.03% to tetracycline at a concentration of 0.016 µg/mL – 256 µg/mL and 83.17% to nalidixic acid at a concentration of 0.016 µg/mL – 256 µg/mL. *Escherichia coli* ATCC 25922 showed susceptibility to all antibiotics used in this study at the above-mentioned concentrations (Table 3).

**TABLE 3:** Susceptibility patterns of *Vibrio cholerae* isolates against commonly used antibiotics to manage cholera (*n* = 101).

Antimicrobial agent	*V. cholerae* isolates				*E. coli* ATCC 25922 (PC)			
	Susceptible		Resistant		Susceptible		Resistant	
	*n*	%	*n*	%	*n*	%	*n*	%
AMX	36	35.64	65	64.36	101	100	00	0.00
AMP	11	10.89	90	86.11	100	99	01	1.00
CRO	100	99.00	01	1.00	101	100	00	0.00
CHL	56	55.45	45	44.55	101	100	00	0.00
CIP	101	100	00	0.00	101	100	00	0.00
SXT	02	1.98	99	98.02	101	100	00	0.00
DOX	100	99.00	01	1.00	101	100	00	0.00
ERY	47	46.53	54	53.47	101	100	00	0.00
GEN	101	100	00	0.00	101	100	00	0.00
NAL	17	16.83	84	83.17	101	100	00	0.00
OFX	101	100	00	0.00	101	100	00	0.00
STR	96	96.04	5	3.96	101	100	00	0.00
TET	5	4.95	96	97.03	101	100	00	0.00
0/129	8	7.93	93	92.07	101	100	00	0.00

AMX, amoxicillin; AMP, ampicillin; CRO, ceftriaxone; CHL, chloramphenicol; CIP, ciprofloxacin; SXT, cotrimoxazole; DOX, doxycycline; ERY, erythromycin; GEN, gentamycin; NAL, nalidixic acid; OFX, ofloxacin; STR, streptomycin; TET, tetracycline; O/129, vibriostatic agent; PC, positive control isolate; *E. coli, Escherichia coli*; *V. cholerae, Vibrio cholerae.*

For Etest, all isolates were susceptible to ciprofloxacin and gentamicin. However, there was a reduced susceptibility to chloramphenicol, with 55.45% of isolates being susceptible and 4.55% were being resistant. The MIC ranged from 0.25 µg/mL to 96 µg/mL. In all, 99% of *V. cholerae* isolates were susceptible to ceftriaxone, 96.04% were susceptible to streptomycin and 99% were susceptible to doxycycline. The MIC50 of ceftriaxone, doxycycline and streptomycin were in the susceptible zone.

It was also deduced that 64.36% of the *V. cholerae* isolates were resistant to amoxicillin (MIC 0.75: > 256 µg/mL), 53.47% to erythromycin (MIC 0.125: 48 µg/mL) and 83.17% to nalidixic acid (MIC 0.125: > 256 µg/mL). The MIC50 of nalidixic acid, amoxicillin and erythromycin were in the resistant zone. These results are in agreement with the results obtained by the disk diffusion method. MIC50 for E. coli standard strain ATCC 25922 was susceptible to all antibiotics used in this study.

### Susceptibility to O/129 vibriostatic agent

The evaluation of susceptibility towards the O/129 vibriostatic agent shows that the majority of the *V. cholerae* isolates (93 out of 101; 92.07%) were resistant to this agent at a concentration of 150 µg, with only 8 (7.93%) being sensitive with an inhibition of ≥ 16 mm (Table 3).

## Discussion

Serological characterisation revealed that the majority of isolates (73.95%) belonged to serotype Inaba. This finding concurs with a previous study conducted on *V. cholerae* O1 strains isolated in the coastal region of Kenya between 2005 and 2007, where serotype Inaba emerged as the main cause of epidemic.^16^ Similar findings were reported in another study carried out on *V. cholerae* O1 strains isolated in Kisumu county in 2014, where serotype Inaba emerged as the main cause of epidemic. These findings suggest a shift in the occurrence of Ogawa and Inaba serotypes in a given area, which is thought to be a consequence of genetic reversal that makes it possible for the Ogawa strain, which was dormant in the environment, to be able to mutate to serotype Inaba.^24^ It appears that as an alternate to Ogawa serotype, Inaba has appeared to aid the persistence of cholera and thus perpetuate the spread of *V. cholerae* El Tor.

The susceptibility patterns of *V. cholerae* isolates showed alarmingly high resistance (97%) towards tetracycline, whose mode of action is reversibly binding to receptors on the 30S ribosomal subunit of bacteria, preventing attachment of aminoacyl-transferable ribonucleic acid (tRNA) to the transferable ribonucleic acid (RNA)-ribosome complex, and this prevents the addition of amino acids to elongating peptide chain, preventing protein synthesis. These findings do not concur with the findings of a previous study that were reported during another outbreak in 2014 in Kisumu county.^24^ These findings reported that 100% of isolates were susceptible to tetracycline, a fact that should be of major concern because there was a gap of hardly 5 years between the two outbreaks. Most probably, this is attributed to the rise of antimicrobial resistance globally, as bacteria now could interchange resistant genes amongst one another because of integron genes which are described as vehicles for the acquisition of antibiotic-resistant genes.^25^ These findings suggest that tetracycline should not be used for the treatment of cholera infections caused by current *V. cholerae* O1 strains. It is thus important to monitor tetracycline-resistant patterns against *V. cholerae* strains, more especially at the study site, because this has been the drug of choice during cholera outbreaks,^26^ and therefore its preferred use could be responsible for reoccurrence of cholera in the region as fewer susceptibility tests were conducted before its administration. This finding concurs with a study performed in Mozambique on antimicrobial resistance of *V. cholerae* O1 isolates in 2007; the study reported high incidence of resistance to tetracycline (97.3%), used as the first-line drug for cholera treatment.^27^ Doxycycline, on the other hand, has been used extensively in this county for many years for cholera management. However, another study conducted in Kenya in 2007 to determine the antimicrobial response of *V. cholerae* isolates reported that all isolates were susceptible to doxycycline.^28^ In the present study, there was less resistance to doxycycline (i.e. only in 1.00% of isolates), suggesting that the antibiotic was effective in managing cholera in Kisumu County; however, close attention should be given to the emergence of these resistant strains.

A high incidence of resistance to cotrimoxazole (98.02%) was also observed in the present study, which is something to worry about. This finding is in agreement with previous studies conducted in Kenya^16^ and Mozambique^27,29^ that reported 97% and 99% resistance, respectively. Cotrimoxazole has been the first-line treatment for diarrhoea infections in Kenya, and it is commonly prescribed not only for gastrointestinal tract infections, including diarrhoea, but also for the treatment of respiratory tract infections, urinary tract infections and skin infections. Its mode of action is by targeting a subunit of DNA gyrase, which is essential in the production of bacteria DNA. It is often prescribed to immunocompromised patients, including human immunodeficiency virus (HIV) patients, which are common in the study area with a prevalence of 16.3%.^13^ Such a vast usage of cotrimoxazole, and the fact that it is relatively cheap and could be acquired over the counter even without a prescription, may have contributed to the emergence of resistance towards this antibiotic as observed in this study.

A high incidence of chloramphenicol-resistant isolates (44.55%) was also observed. Chloramphenicol is a bacteriostatic and broad-spectrum antibiotic effective against a wide variety of Gram-positive and Gram-negative bacteria. Its mode of action is by irreversibly binding to a receptor site on the 50S subunit of bacterial ribosome, inhibiting peptidyl transferase, consequently resulting in the prevention of amino acid transfer to growing peptide chains, leading to inhibition of protein synthesis. Up to the late 1990s, it was used as the first-line antibiotic treatment for typhoid and other *Salmonella* infections.^30^ However, because of resistance and safety issues, it is no longer the first-line treatment in enteritis. In low-income countries, it is still widely used, as it is not expensive and readily available.^29^ Chloramphenicol has been recommended by the World Health Organization (WHO) for the treatment of cholera in children and pregnant women.^31^ The high resistance observed may be explained by its frequent usage for the treatment of severe diarrhoea and other infectious diseases. Other reports in Kenya have indicated that *V. cholerae* O1 isolates are resistant to chloramphenicol.^16^. A similar incidence was observed for other pathogens causing diarrhoea in a study conducted in the study area.^31^

A higher resistance rate to ampicillin (89.11%) was observed compared to that towards amoxicillin (64.36%), whose mode of activity is by targeting penicillin-binding proteins – a group of enzymes found anchored in the cell membrane, involved in the cross-linking of bacterial cell wall. Cholera isolates from previous outbreaks in Kenya were known to exhibit resistance to ampicillin,^13^ doxycycline and streptomycin.^32^ There was an emergence of isolates resistant to nalidixic acid as well, with 81% resistant to this antimicrobial which has been primarily associated with the presence of IncC conjugative plasmids.^30^ All isolates were sensitive towards ciprofloxacin, gentamicin and ofloxacin, thus confirming the higher efficiency of these agents against *V. cholerae* isolates at Kisumu County.

These findings therefore clearly demonstrate the need for susceptibility tests to be carried out as some resistance cases of *V. cholerae* to common antibiotics used to manage enteritis and diarrhoea have been documented. The findings also demonstrate that it is high time that the usage of tetracycline and cotrimoxazole needs to be monitored as their high resistance levels are of great concern.
